# Anatomy of a Cohort: 40-Year Follow-Up of a Sjögren’s Cohort

**DOI:** 10.3390/jcm15093316

**Published:** 2026-04-27

**Authors:** Blanca Viejo-Sosa, Uxía Couto-Lareo, Mònica Angerri-Nadal, David A. Isenberg

**Affiliations:** 1 Servicio de Reumatología, Hospital Universitario Jerez de la Frontera, 11407 Cádiz, Spain; bvsosa@gmail.com; 2 Servicio de Reumatología, Hospital Universitario A Coruña, 15006 A Coruña, Spain; ucoula@gmail.com; 3 Servei Medicina Interna, Hospital Universitari Dr. Josep Trueta, 17007 Girona, Spain; mangerri.girona.ics@gencat.cat; 4 Department of Ageing, Rheumatology and Regenerative Medicine, University College London, London WC1E 6BT, UK

**Keywords:** Sjogren’s Disease, serological pattern, long-term follow-up, ethnic groups, lymphoma

## Abstract

**Background**: Sjögren’s disease (SjD) is a chronic autoimmune rheumatic disorder primarily affecting exocrine glands, leading to dryness and systemic involvement. B-cell hyperactivity and autoantibody production drive its pathogenesis and contribute to increased lymphoma risk. Although several long-term studies exist, we present a review of a closely monitored cohort assessed over 40 years. **Methods**: Retrospective observational study at University College London Hospital included patients fulfilling the 2016 ACR/EULAR criteria for SjD between 1986–2025. Patients with associated SjD were excluded. Associations between serological markers and clinical features were analysed using chi-square or Fisher’s exact tests (*p* < 0.05). Differences between ethnic groups were also assessed. **Results:** 283 patients were included, 93.3% female, with mean age at diagnosis of 50.1 ± 15.2 years and mean follow-up of 12.5 ± 8.6 years. Common manifestations were fatigue (61.5%), parotid swelling (30.5%), arthritis (25.8%), and Raynaud’s phenomenon (27.6%). Anti-Ro and anti-La antibodies were present in 75.7% and 45.2%, respectively; rheumatoid factor in 57.3%. Lymphoma developed in 9.9% (mostly non-Hodgkin MALT) and was associated with hypergammaglobulinemia (*p* = 0.03; RR = 2.56) and parotid swelling (*p* < 0.001; RR = 5.53). Serological markers correlated with systemic features including lymphadenopathy, vasculitis, and pulmonary involvement. Caucasian patients showed higher mortality (*p* < 0.001; RR = 3.89) and peripheral nervous system involvement (*p* = 0.02; RR = 2.18), and less ANA positivity (*p* = 0.004; RR = 0.88), anti-Ro (*p* = <0.001; RR = 0.77) and RF (*p* = 0.04; RR = 0.81) and hypergammaglobulinemia (*p* = <0.001; RR = 0.63) when compared with non-Caucasian patients. **Conclusions:** This long-term cohort confirms the strong association between B-cell activation markers and adverse outcomes in Sjögren’s disease. Hypergammaglobulinemia and parotid swelling emerged as key predictors of lymphoma, supporting their role in risk stratification. These findings reinforce the importance of long-term monitoring and may help guide personalized clinical management and surveillance strategies.

## 1. Introduction

Sjögren’s disease (SjD) is a chronic autoimmune rheumatic disorder characterized by lymphocytic infiltration of exocrine glands, leading to sicca symptoms and systemic manifestations in 30–40% patients. The disease shows marked clinical heterogeneity, often resulting in diagnostic delays [1]. SjD occurs alone, or with organ-specific autoimmune disorders such as thyroiditis, whereas associated SjD (formerly associated) coexists with systemic diseases like systemic lupus erythematosus or rheumatoid arthritis [2]. The 2016 ACR/EULAR classification criteria provide a standardized framework with high sensitivity and specificity [3].

The estimated prevalence of SjD ranges from 0.3 to 1 per 1000 individuals, predominantly affecting women aged 50–70 years [4,5]. Its pathogenesis is multifactorial, involving genetic, hormonal, and environmental factors. Salivary gland epithelial cells promote local immune activation by recruiting inflammatory cells [6]. Central to pathogenesis is B-cell hyperactivity, which drives autoantibody production, hypergammaglobulinemia, and lymphoepithelial lesion formation [6]. These features ultimately predispose to mucosa-associated lymphoid tissue (MALT) lymphoma [7].

Clinically, SjD key symptoms are xerostomia and xerophthalmia present in more than 90% of patients [4]. It may manifest with fatigue, arthralgia, and salivary gland swelling [8,9,10]. Extraglandular involvement may include pulmonary, neurological, renal, hepatic, and cutaneous features [11,12,13,14,15,16,17]. Serologically, the most characteristic markers are anti-Ro/SSA and anti-La/SSB antibodies [4,18], often accompanied by anti-nuclear antibodies [ANA] (positive in up to 80%), rheumatoid factor [RF] (up to 50%), and complement consumption (hypocomplementemia in up to 5%) [19]. Some of these autoantibodies may be present for 20 years before the onset of the symptoms [20] and are associated with more severe phenotypes and poorer prognosis. 

The main causes of death in SjD are neoplasms (including hematological and solid organ cancers), infections, and cardiovascular disease [21,22]. Patients with SjD have a 10- to 44-fold increased risk of lymphoma compared with the general population [23,24], mainly mucosa-associated lymphoid tissue (MALT) and low-grade B-cell non-Hodgkin lymphomas [25]. Furthermore, a higher risk of solid tumors, especially thyroid, gastric, and oral cancers, has also been described [26,27].

Although many studies have explored outcomes in SjD, disease classification and long-term prognosis remain uncertain. Most cohorts have relatively short follow-up, with very few exceeding ten years.

In an earlier study, we reported 152 patients monitored for up to 25 years (mean 12.5 years), showing that SjD, though often considered benign, can cause serious complications and reduced quality of life [28]. In Sweden, a large cohort of 8884 patients was followed for an average of 6.6 years, focusing mainly on productivity loss and healthcare costs [29]. A national Chinese cohort of 1.054 patients, followed for nearly eight years, reported higher mortality compared with the general population, with infections and solid-organ malignancies as leading causes of death, while overall cancer risk, particularly for lymphoma, was lower than in Western populations [30].

These findings emphasize the importance of long-term observational research to better define optimally the clinical course, comorbidities, and survival outcomes in SjD.

We present the longest follow-up, single center cohort of SjD patients that has been described in the literature. This cohort was established in 1986 and has 40 years of follow-up [17,19,31]. The main objective is to analyze the association between clinical and serological phenotypes and long-term outcomes, especially the development of lymphoma [32]. We also sought to determine if there were any differences in ethnicity comparing our Caucasian with non Caucasian patients [24,33].

## 2. Materials and Methods

### 2.1. Study Design

A retrospective, very long-term observational study of patients diagnosed with SjD at University College London Hospital (UCLH) was performed. Demographic, clinical, and serological data were extracted from paper and electronic health records collected over a 40-year period. The study was performed in accordance with the ethical standards stated in the Declaration of Helsinki.

### 2.2. Data Collection

We included patients who fulfilled with SjD 2016 EULAR/ACR criteria, with follow-up appointments 6 months on average in the UCLH Sjogren’s Clinic between 1986 and 2025. Those who had associated SjD were excluded.

Demographic variables collected included age at diagnosis, gender, ethnicity, and follow-up duration (Table 1). Clinical variable features included glandular manifestations (parotid swelling, lymphadenopathy), systemic features (arthritis, Raynaud’s phenomenon, fatigue), mucocutaneous manifestations (rash, ulcers, vasculitis), and organ involvement (lung, liver, central nervous system [CNS]—that included optic neuritis and transverse myelitis, peripheral nervous system [PNS]—including carpal tunnel syndrome, mononeuropathy, polyneuropathy). Histological confirmation by minor salivary gland biopsy and imaging studies (ultrasound of salivary glands) were recorded.

Serological assessments included antinuclear antibodies (ANA) with pattern description if available, anti-Ro/SS-A and anti-La/SS-B antibodies, rheumatoid factor (RF), anti-RNP antibodies, complement levels (C3, C4), and hypergammaglobulinemia. Laboratory data were collected at baseline or at the earliest available timepoint, as longitudinal measurements were not consistently available for all patients. Lymphoma and other malignancy diagnoses were recorded, including lymphoma subtypes and time from SjD diagnosis to lymphoma development.

Other autoimmune disorders which were diagnosed at least one year after SjD diagnosis were recorded. These included thyroid (hypo and hyperthyroidism confirmed as autoimmune, autoimmune thyroiditis), dermatological (cutaneous lupus, psoriasis, vitiligo), gastrointestinal (celiac disease, pernicious anemia, ulcerative colitis).

A number of patients were lost to follow-up (some moved away, some very elderly who found it difficult to keep clinic appointments). For long-term follow-up purposes, we tried very hard to contact patients in this category to determine if they were still alive, and if they had died, what the cause of death was.

### 2.3. Statistical Analysis

Descriptive statistics were used for demographic and clinical characteristics. Continuous variables are presented as mean ± standard deviation (SD), and categorical variables as frequencies and percentages. Associations between clinical manifestations and serological markers (anti-Ro, anti-La, RF, hypergammaglobulinemia) and lymphoma or ethnicity were evaluated using Chi-square or Fisher’s exact test, as appropriate. Relative risks (RRs) with 95% confidence intervals (CI) were calculated for significant associations. Combined serological profiles were analyzed to explore overlapping risk patterns. *p*-value < 0.05 was considered statistically significant. To account for multiple comparisons in Table 2 and Table 3, *p*-values were adjusted using the Holm–Bonferroni method.

Missing data were handled using an available-case analysis approach, and the number of patients included in each analysis is specified for each variable. No imputation methods were applied.

Given the exploratory nature of this study and the large number of comparisons performed, no formal adjustment for multiple testing was applied. Therefore, the results should be interpreted with caution, particularly for marginally significant associations.

## 3. Results

### 3.1. Demographic, Clinical and Serological Charactersitics of UCLH Cohort

Baseline demographic, clinical, and serological characteristics of the UCLH cohort are summarized in Table 1.

A total of 283 patients were included, of whom 93.3% (264) were female. The mean age at diagnosis was 50.1 ± 15.2 years. The predominant ethnic group was Caucasian (60.4%), followed by South Asian (15.2%), other ethnicities (13.4%), Black (8.8%), and mixed (2.1%). The mean follow-up duration was 12.5 ± 8.6 years.

A total of 96 patients (33.9%) were lost to follow-up, of whom 48 (50%) had died. The overall mortality rate was 18.6%. The main causes of death were infection (34.4%), lymphoma (12.5%), and other cancers (6.3%), while the cause remained unknown in 21.9% of cases.

Fatigue was the most frequent clinical manifestation, reported in 61.5% of patients. Among glandular features, the most common manifestation was parotid gland swelling (30.5%). Lymphadenopathy was present in 14.9%, arthritis in 25.8%, and Raynaud’s phenomenon in 27.6%. Cutaneous rash occurred in 24.7%, while ulcers and vasculitis were observed in 8.5% and 8.5%, respectively.

Among systemic manifestations, pulmonary involvement was present in 6.7% of patients, including interstitial lung disease (4.8%) and pulmonary hypertension (0.7%). Central nervous system (CNS) and peripheral nervous system (PNS) involvement were observed in 1.5% and 14.1% of patients, respectively. Renal involvement was identified in 2.6%, mainly renal tubular acidosis, and hepatic involvement in 5.9%.

Serologically, ANA positivity was found in 87.4% of patients, most commonly with a speckled pattern. Anti-Ro antibodies were present in 75.7% and anti-La antibodies in 45.2%, while rheumatoid factor (RF) was positive in 57.3%. Hypergammaglobulinemia was observed in 52.5% of patients.

Lymphoma developed in 9.9% of patients, predominantly of the non-Hodgkin type, with mucosa-associated lymphoid tissue (MALT) lymphoma accounting for 73.1% of cases. Most lymphomas occurred within the first 15 years following Sjögren’s disease diagnosis.

**Table 1:** Baseline demographic, clinical, and serological characteristics of the UCLH cohort. This table summarizes the baseline demographic, clinical, and serological characteristics of patients included in the UCLH cohort (n = 283). Categorical variables are presented as absolute numbers and corresponding percentages, while continuous variables are shown as mean ± standard deviation (SD). Percentages were calculated based on the total number of patients for whom data were available for each variable.

	UCL Cohort (n = 283)
**Epidemiology**	
Gender (female)	264/283 (93.3%)
Age at diagnosis	50.1 ± 15.2 (SD)
**Clinical involvement**	
Parotid swelling	82/269 (30.5%)
Lymphadenopathy	40/269 (14.9%)
Arthritis	73/283 (25.8%)
Fatigue	174/283 (61.5%)
Raynaud	78/283 (27.6%)
Ulcers	40/283 (8.5%)
Vasculitis	24/283 (8.5%)
Rash	70/283 (24.7%)
**Serology**	
(+) Anti-Ro/SSA	206/272 (75.7%)
(+) Anti-La/SSB	123/272 (45.2%)
(+) ANA	235/269 (87.4%)
Sparkled	195/233 (83.7%)
Homogeneous	18/233 (7.7%)
Nucleolar	5/233 (2.1%)
Centromeric	6/233 (2.6%)
Dotts	8/233 (3.4%)
(+) RF	150/262 (57.3%)
(+) Anti-RNP	10/272 (3.7%)
Low complement	31/247 (12.6%)
Low C3	10/247 (4.0%)
Low C4	11/247 (4.5%)
Both Low	7/247 (2.8%)
Hypergammaglobulinemia	136/259 (52.5%)
**Lymphoma**	26/283 (9.9%)
Hodgkin	1/26 (3.8%)
Non-Hodgkin	25/26 (96.2%)
MALT	19/26 (73.1%)
Others	6/26 (23.1%)
Lymphoma develop <15 years of SjD	19/24 (79.2%)
**Other Cancers**	41/283 (14.5%)
**Lost to follow-up**	96/283 (33.9%)
Dead	48/96 (50%)
**Death**	48/258 (18.6%)
Infection	11/48 (34.4%)
Other	8/48 (25.0%)
Unknown	7/48 (21.9%)
Lymphoma	4/48 (12.5%)
Cancer	2/48 (6.3%)

### 3.2. Association Between Serological Markers and Clinical Manifestations

The associations between serological markers (anti-Ro, anti-La, rheumatoid factor [RF] and hypergammaglobulinemia) with clinical features are shown in Table 2. Associations between serological markers and clinical manifestations were analyzed using group-based comparisons, with proportions calculated within each serological group.

Among anti-Ro-positive patients, parotid swelling was observed in 35.4% compared with 10.6% of anti-Ro-negative patients (*p* < 0.001; adjusted *p* < 0.001). Lymphadenopathy was more frequent in anti-Ro-positive patients (17.5% vs. 6.1%), although this association did not remain statistically significant after Holm–Bonferroni correction (adjusted *p* = 0.076).

Among anti-La-positive patients, both parotid swelling (37.4% vs. 22.8%; adjusted *p* = 0.038) and lymphadenopathy (21.1% vs. 9.4%; adjusted *p* = 0.033) remained significantly associated after correction for multiple comparisons.

Patients with hypergammaglobulinemia showed significantly higher frequencies of parotid swelling (39.0% vs. 20.3%; adjusted *p* = 0.004) and lymphadenopathy (21.3% vs. 7.3%; adjusted *p* = 0.006).

Although rheumatoid factor positivity was associated with higher frequencies of several clinical manifestations in unadjusted analyses, none of these associations remained statistically significant after correction for multiple testing.

No significant associations were observed between serological markers and fatigue or arthritis after adjustment for multiple comparisons.

### 3.3. Association Between Combined Serological Profiles and Clinical Manifestations

In Table 3, a combined analysis of the serology items was performed. Combined serological profiles were further analyzed to explore overlapping risk patterns.

Patients positive for both anti-Ro and anti-La antibodies showed a higher prevalence of lung involvement (10.6% vs. 2.0%; adjusted *p* = 0.036), which remained statistically significant after correction for multiple comparisons. Other associations, including parotid swelling and lymphadenopathy, did not remain significant after adjustment.

Patients with triple seropositivity (anti-Ro, anti-La, and RF) demonstrated a broader systemic phenotype. In this group, lymphadenopathy (25.3% vs. 9.7%; adjusted *p* = 0.018), vasculitis (16.1% vs. 4.3%; adjusted *p* = 0.009), and lung involvement (16.1% vs. 3.8%; adjusted *p* = 0.009) were significantly more frequent compared with other patients.

No significant differences were observed for arthritis, fatigue, Raynaud’s phenomenon, hepatic involvement, CNS involvement, or PNS involvement after correction for multiple testing.

### 3.4. Comparing Caucasian with Non-Caucasian

Table 4 compares differences between clinical, serological, and lymphoma development in both groups.

Among the cohort, 60.4% of patients were Caucasian. The proportion of females was similar between Caucasian and non-Caucasian groups (93.6% vs. 92.9%).

Caucasian patients had a significantly higher mortality rate compared with non-Caucasian patients (26.5% vs. 6.8%, *p* < 0.001; RR = 3.89). Loss to follow-up was also more frequent in Caucasian patients (43.3% vs. 19.6%, *p* < 0.001; RR = 2.20).

**Table 2:** Association between clinical manifestations and serological/analytical markers. Results are expressed as the percentage of patients with each clinical manifestation among those positive or negative corresponding serological markers, together with the absolute number of cases (n). For each association, the corresponding *p*-value (Chi-square or Fisher’s exact test, as appropriate) and the relative risk (RR, 95% CI) are reported. NA indicates values not applicable or not calculable due to zero frequencies. Variables that demonstrate statistical significance are highlighted in bold.

Variable	Anti-Ro (+) (n = 206)	Anti-Ro (−) (n = 66)	*p*-Value	Adj *p*-Value	Anti-La (+) (n = 123)	Anti-La (−) (n = 149)	*p*-Value	Adj *p*-Value	RF (+) (n = 150)	RF (−) (n = 112)	*p*-Value	Adj *p*-Value	HGG (+) (n = 136)	HGG (−) (n = 123)	*p*-Value	Adj *p*-Value
Parotid swelling	73/206 (35.4%)	7/66 (10.6%)	**<0.001**	**<0.001**	46/123 (37.4%)	34/149 (22.8%)	**0.013**	**0.038**	53/150 (35.3%)	27/112 (24.1%)	0.050	0.10	53/136 (39.0%)	25/123 (20.3%)	**0.001**	**0.004**
Lymphadenopathy	36/206 (17.5%)	4/66 (6.1%)	**0.038**	0.076	26/123 (21.1%)	14/149 (9.4%)	**0.011**	**0.033**	29/150 (19.3%)	11/112 (9.8%)	0.040	0.080	29/136 (21.3%)	9/123 (7.3%)	**0.002**	**0.006**
Arthritis	54/206 (26.2%)	15/66 (22.7%)	0.69	0.69	33/123 (26.8%)	36/149 (24.2%)	0.72	0.72	46/150 (30.7%)	22/112 (19.6%)	0.044	0.088	39/136 (28.7%)	23/123 (18.7%)	0.060	0.12
Fatigue	125/206 (60.7%)	42/66 (63.6%)	0.78	0.78	75/123 (61.0%)	92/149 (61.7%)	1.00	1.00	90/150 (60.0%)	71/112 (63.4%)	0.58	0.58	84/136 (61.8%)	75/123 (61.0%)	0.90	0.90
Raynaud	47/206 (22.8%)	26/66 (39.4%)	**0.008**	**0.032**	29/123 (23.6%)	44/149 (29.5%)	0.27	0.54	41/150 (27.3%)	28/112 (25.0%)	0.67	0.80	30/136 (22.1%)	38/123 (30.9%)	0.11	0.22
Mouth ulcers	27/206 (13.1%)	10/66 (15.2%)	0.67	0.80	18/123 (14.6%)	19/149 (12.8%)	0.65	0.80	30/150 (20.0%)	6/112 (5.4%)	**<0.001**	**0.004**	20/136 (14.7%)	12/123 (9.8%)	0.23	0.46
Vasculitis	22/206 (10.7%)	0/66 (0.0%)	**0.003**	**0.012**	16/123 (13.0%)	6/149 (4.0%)	**0.007**	**0.028**	17/150 (11.3%)	4/112 (3.6%)	**0.023**	**0.069**	17/136 (12.5%)	5/123 (4.1%)	**0.030**	**0.090**
Lung involvement	15/206 (7.3%)	1/66 (1.5%)	0.13	0.26	13/123 (10.6%)	3/149 (2.0%)	**0.004**	**0.016**	11/150 (7.3%)	5/112 (4.5%)	0.44	0.66	13/136 (9.6%)	3/123 (2.4%)	**0.020**	**0.080**
Liver involvement	13/206 (6.3%)	3/66 (4.5%)	0.77	0.88	6/123 (4.9%)	10/149 (6.7%)	0.52	0.78	8/150 (5.3%)	8/112 (7.1%)	0.55	0.78	11/136 (8.1%)	4/123 (3.3%)	0.12	0.36
CNS	1/206 (0.5%)	2/66 (3.0%)	0.15	0.30	1/123 (0.8%)	2/149 (1.3%)	1.00	1.00	0/150 (0.0%)	3/112 (2.7%)	0.08	0.24	0/136 (0.0%)	4/123 (3.3%)	**0.05**	0.15
PNS	24/206 (11.7%)	12/66 (18.2%)	0.17	0.34	9/123 (7.3%)	27/149 (18.1%)	**0.009**	**0.036**	14/150 (9.3%)	22/112 (19.6%)	**0.016**	**0.048**	12/136 (8.8%)	23/123 (18.7%)	**0.020**	0.060

**Table 3:** Association between clinical manifestations and serological-combined markers. Results are expressed as the percentage of patients with each clinical manifestation among those positive or negative corresponding serological marker, together with the absolute number of cases (n). For each association, the corresponding *p*-value (Chi-square or Fisher’s exact test, as appropriate) and the relative risk (RR, 95% CI) are reported. NA indicates values not applicable or not calculable due to zero frequencies. Variables that demonstrate statistical significance are highlighted in bold.

Variable	Ro + La+ (n = 123)	Others (n = 149)	*p*-Value	Adj *p*-Value	Ro + La + RF+ (n = 87)	Others (n = 185)	*p*-Value	Adj *p*-Value
Parotid swelling	46/123 (37.4%)	34/149 (22.8%)	**0.013**	**0.078**	33/87 (37.9%)	47/185 (25.4%)	0.05	0.40
Lymphadenopathy	26/123 (21.1%)	14/149 (9.4%)	**0.011**	**0.077**	22/87 (25.3%)	18/185 (9.7%)	**0.002**	**0.018**
Arthritis	33/123 (26.8%)	36/149 (24.2%)	0.72	1.00	26/87 (29.9%)	43/185 (23.2%)	0.27	1.00
Fatigue	75/123 (61.0%)	92/149 (61.7%)	1.00	1.00	50/87 (57.5%)	117/185 (63.2%)	0.37	1.00
Raynaud	29/123 (23.6%)	44/149 (29.5%)	0.27	1.00	23/87 (26.4%)	50/185 (27.0%)	0.92	1.00
Vasculitis	16/123 (13.0%)	6/149 (4.0%)	**0.007**	**0.056**	14/87 (16.1%)	8/185 (4.3%)	**0.001**	**0.009**
Lung involvement	13/123 (10.6%)	3/149 (2.0%)	**0.004**	**0.036**	14/87 (16.1%)	7/185 (3.8%)	**0.001**	**0.009**
Liver involvement	6/123 (4.9%)	10/149 (6.7%)	0.52	1.00	10/87 (11.5%)	6/185 (3.2%)	0.04	0.32
CNS	1/123 (0.8%)	2/149 (1.3%)	1.00	1.00	3/87 (3.4%)	12/185 (6.5%)	0.55	1.00
PNS	9/123 (7.3%)	27/149 (18.1%)	**0.009**	**0.072**	0/87 (0%)	36/185 (19.5%)	**0.022**	0.176
PNS	9/123 (7.3%)	27/149 (18.1%)	**0.009**	**0.072**	0/87 (0%)	36/185 (19.5%)	**0.022**	0.176

In terms of clinical manifestations, Caucasian patients showed a lower prevalence of parotid swelling (*p* = 0.002; RR = 0.57) and a higher prevalence of peripheral nervous system involvement (*p* = 0.02; RR = 2.18). No other clinical features differed significantly between groups.

Serologically, Caucasian patients had lower frequencies of ANA positivity (*p* = 0.004), anti-Ro antibodies (*p* < 0.001), RF positivity (*p* = 0.04), and hypergammaglobulinemia (*p* < 0.001) compared with non-Caucasian patients. No significant differences were observed for anti-La antibodies or combined serological profiles.

No statistically significant differences were found in lymphoma prevalence between the two groups.

**Table 4:** Comparing Caucasian with non-Caucasian patients. Results are expressed as the percentage (%) of patients with each manifestation in the Caucasian and non-Caucasian group, together with the relative and absolute number of cases (n/n). For each association, the corresponding *p*-value (* Chi-square or Fisher’s exact test, as appropriate) and the relative risk (RR, 95% CI) are reported. Variables that demonstrate statistical significance are highlighted in bold.

	Caucasian	Non-Caucasian	Adjusted *p*-Value (RR) * Fisher Exact Test
Death	26.5% (41/155)	6.8% (7/103)	***p* = <0.001** RR = 3.89 (1.82–8.34)
Lost to Follow-up	43.3% (74/171)	19.6% (22/112)	***p* = <0.001** RR = 2.20 (1.46–3.33)
Parotid swelling	23.5% (38/162)	41.4% (44/107)	***p* = 0.002** RR = 0.57 (0.40–0.82)
Lymphadenopathy	13.0% (21/161)	17.6% (19/108)	*p* = 0.30
Arthritis	25.7% (44/171)	25.9% (29/112)	*p* = 0.98
Fatigue	63.2% (108/171)	59.8% (67/112)	*p* = 0.57
Raynaud	31.0% (53/171)	22.3% (25/112)	*p* = 0.11
Mouth ulcers	15.8% (27/171)	11.6% (13/112)	*p* = 0.32
Vasculitis	9.4% (16/171)	7.1% (8/112)	*p* = 0.51
Liver impairment	6.4% (11/171)	5.4% (6/112)	*p* = 0.71
Lung impairment	7.0% (12/171)	6.3% (7/112)	*p* = 0.80
CNS	1.8% (3/171)	0.9% (1/111)	*p* = 1 *
PNS	17.5% (30/171)	8.0% (9/112)	***p* = 0.02** RR = 2.18 (1.08–4.42)
ANA-positive	82.6% (133/161)	94.4% (102/108)	***p* = 0.004** RR = 0.88 (0.81–0.95)
Ro-positive	67.7% (111/164)	88.0% (95/108)	***p* = <0.001** RR = 0.77 (0.68–0.87)
La-positive	40.9% (67/164)	51.9% (56/108)	*p* = 0.08
RF-positive	52.2% (82/157)	64.8% (68/105)	***p* = 0.04** RR = 0.81 (0.66–0.99)
Hypergammaglobulinemia	42.6% (66/155)	67.3% (70/104)	***p* = <0.001** RR = 0.63 (0.50–0.79)
Ro- and La-positive	40.9% (67/164)	51.9% (56/108)	*p* = 0.075
Ro- and La- and RF-positive	31.2% (49/157)	36.5% (38/104)	*p* = 0.371
Lymphoma	9.9% (17/171)	9.8% (11/112)	*p* = 0.97

### 3.5. Lymphoma Risk

Table 5 summarizes the prevalence of lymphoma in relation to various clinical and serological features. Lymphoma developed in 9.9% of patients.

Hypergammaglobulinemia and parotid swelling were significantly associated with lymphoma development. Hypergammaglobulinemia was present in 73.9% of lymphoma cases (*p* = 0.03; RR = 2.56), while parotid swelling was observed in 70.8% of patients with lymphoma (*p* < 0.001; RR = 5.53).

Other serological markers, including anti-Ro, anti-La, and RF, were not significantly associated with lymphoma. Similarly, no significant associations were observed for lymphadenopathy, arthritis, Raynaud’s phenomenon, mucocutaneous manifestations, lung involvement, or other autoimmune conditions.

These findings highlight the role of chronic B-cell activation and glandular involvement as key predictors of lymphoma development in this cohort.

**Table 5:** Clinical and serological characteristics associated with lymphoma in the UCLH cohort. Results are presented as the percentage and number (n) of lymphoma cases among patients with each feature. * *p*-values were calculated using Chi-square or Fisher ex.

Variable		Lymphoma % (n)	Adjusted *p*-Value * Fisher Exact Test
Ro	+	88.0% (22)	0.15 *
	−	12.0% (3)	
La	+	52.0% (13)	0.48
	−	48.0% (12)	
Ro and La	+	52.0% (13)	0.48
	−	48.0% (12)	
RF	+	75.0% (18)	**0.07**
	−	25.0% (6)	
Ro, La and RF	+	43.5% (10)	0.28
	−	56.5% (13)	
Hypergammaglobulinemia	Yes	73.9% (17)	**0.03** RR = 2.56 (1.04–6.28)
	No	26.1% (6)	
Parotid swelling	Yes	70.8% (17)	**<0.001** RR = 5.53 (2.39–12.85)
	No	29.2% (7)	
Lymphadenopathy	Yes	25.0% (6)	0.14
	No	75.0% (18)	
Arthritis	Yes	28.6% (8)	0.72
	No	71.4% (20)	
Raynaud	Yes	25.0% (7)	0.75
	No	75.0% (21)	
Mouth ulcers	Yes	14.3% (4)	1.0 *
	No	85.7% (24)	
Vasculitis	Yes	14.3% (4)	0.28 *
	No	85.7% (24)	
Lung	Yes	14.3% (4)	0.11 *
	No	85.7% (24)	
Other Autoimmune	Yes	25.0% (7)	0.21
	No	75.0% (21)	

## 4. Discussion

This single-centre cohort provides a comprehensive, very long-term overview of the demographic, clinical, and serological characteristics of patients with Sjögren’s disease (SjD) managed at a tertiary referral hospital. To our knowledge, this represents one of the longest follow-up cohorts reported to date, allowing for the assessment of long-term outcomes and clinically relevant complications, particularly lymphoma.

The demographic and clinical profile of our cohort is consistent with previously reported series, with a marked female predominance (93.3%) and a mean age at diagnosis of approximately 50 years. The prevalence of glandular and systemic manifestations also aligns with the existing literature, although some differences are observed. Fatigue was the most frequent clinical manifestation in our cohort, affecting more than 60% of patients, reinforcing its importance as a major contributor to disease burden. Among glandular features, parotid swelling was the most common manifestation, reflecting the chronic inflammatory involvement of exocrine glands characteristic of SjD.

When compared with other cohorts, the prevalence of systemic manifestations such as arthritis and Raynaud’s phenomenon was within the expected range, although pulmonary involvement was less frequent in our cohort than in previously published studies. These differences may be related to variations in cohort composition, referral patterns, or definitions of organ involvement.

Serologically, our findings confirm the high prevalence of ANA, anti-Ro, and anti-La antibodies in SjD, consistent with prior studies. Hypergammaglobulinemia was also common, reflecting the central role of B-cell hyperactivity in disease pathogenesis. However, an important contribution of this study is the refined analysis of the relationship between serological markers and clinical manifestations after appropriate statistical correction for multiple comparisons.

In unadjusted analyses, several associations were observed between serological markers and clinical features. However, after applying the Holm–Bonferroni correction, only a limited number of associations remained statistically significant. Parotid swelling was strongly associated with anti-Ro positivity, anti-La positivity, and hypergammaglobulinemia, suggesting that this clinical feature may represent a marker of underlying B-cell-driven disease activity. Similarly, lymphadenopathy remained significantly associated with anti-La positivity and hypergammaglobulinemia, further supporting the link between serological activity and lymphoproliferative features.

Importantly, many associations observed in the initial analysis, particularly those involving rheumatoid factor, did not remain significant after correction for multiple testing. This highlights the importance of cautious interpretation of unadjusted results and underscores the added value of rigorous statistical approaches in avoiding false-positive findings. Our results therefore provide a more conservative but likely more accurate estimate of clinically relevant associations.

The analysis of combined serological profiles offers further insight into disease heterogeneity. Patients with both anti-Ro and anti-La positivity showed an increased prevalence of pulmonary involvement, suggesting that this serological profile may be associated with a more systemic disease phenotype. Moreover, patients with triple seropositivity (anti-Ro, anti-La, and RF) demonstrated a broader pattern of systemic involvement, including lymphadenopathy, vasculitis, and pulmonary disease, even after adjustment for multiple comparisons. These findings support the concept that combined serological profiles may better capture disease severity than individual markers alone.

Ethnic differences observed in our cohort are also noteworthy. Caucasian patients showed higher mortality and higher rates of peripheral nervous system involvement, while non-Caucasian patients exhibited higher frequencies of serological markers, including ANA, anti-Ro, RF, and hypergammaglobulinemia. These findings are consistent with previous studies suggesting that ethnicity may influence disease expression and immunological profile. However, the reasons underlying these differences remain unclear and may involve genetic, environmental, and healthcare-related factors.

Lymphoma prevalence in our cohort was 9.9%, predominantly of the non-Hodgkin MALT type, in line with previous reports. Among the variables analyzed, hypergammaglobulinemia and parotid swelling emerged as significant predictors of lymphoma. These findings reinforce the role of chronic B-cell activation and persistent glandular inflammation as key drivers of lymphomagenesis in SjD. In contrast, other serological markers such as anti-Ro, anti-La, and RF were not significantly associated with lymphoma development, highlighting the need to distinguish between markers of general immune activation and those specifically linked to malignant transformation.

Our study has several strengths. It represents one of the longest follow-up cohorts of SjD patients reported in the literature and includes a relatively large sample size with ethnic diversity. Additionally, the use of standardized classification criteria and a detailed clinical dataset enhances the reliability of our findings. Importantly, the application of statistical correction for multiple comparisons provides a more robust interpretation of the associations observed.

However, several limitations should be acknowledged. This is a single-centre retrospective study, which may limit generalizability. A substantial proportion of patients were lost to follow-up, which may introduce bias, particularly in long-term outcome analyses. In addition, no adjustment for multiple comparisons was performed in some exploratory analyses, which may increase the risk of type I error. Another important limitation is that serological and immunological data were collected only at baseline or at the earliest available time point. Due to the retrospective nature of the study and the extended follow-up period, longitudinal measurements were not consistently available for all patients. As a result, dynamic changes in immune and inflammatory markers over time could not be assessed.

This limitation restricts the ability to explore temporal relationships and prevents establishing causal associations between serological markers and clinical outcomes, including lymphoma development and organ involvement. Furthermore, it limits the potential predictive value of these markers for long-term prognosis.

In addition, no multivariate analyses were performed, which limits the ability to identify independent predictors of outcomes and to control for potential confounding factors.

Future studies should adopt prospective longitudinal designs with repeated measurements of key biomarkers to better define their dynamic role in disease progression and prognosis.

In conclusion, this long-term cohort study confirms the central role of B-cell activation in the clinical expression of Sjögren’s disease and highlights the importance of combining serological and clinical parameters for risk stratification. Hypergammaglobulinemia and parotid swelling emerged as key predictors of lymphoma, while combined serological profiles were associated with more systemic disease phenotypes. These findings may help guide personalized monitoring strategies and identify patients at higher risk of severe complications. Future studies should focus on validating these observations and developing predictive models incorporating clinical, serological, and possibly molecular biomarkers.

## 5. Conclusions

This study represents one of the longest single-centre follow-up cohorts of patients with Sjögren’s disease. Our findings confirm the strong association between specific serological markers and systemic disease expression. In particular, hypergammaglobulinemia and parotid swelling were identified as significant predictors of lymphoma development.

These results support the clinical utility of combining serological and clinical features to improve risk stratification and patient monitoring. Furthermore, the observed differences between ethnic groups highlight the potential influence of demographic factors on disease phenotype and outcomes.

Future research should focus on validating predictive models incorporating these variables and on developing targeted surveillance strategies to improve early detection of complications, particularly lymphoma.

## Data Availability

The data presented in this study are available on request from the corresponding author.

## Abbreviations

The following abbreviations are used in this manuscript:

SjD	Sjögren’s disease
MALT	Mucosa-Associated Lymphoid Tissue Lymphoma
ANA	anti-nuclear antibodies
RF	Rheumatoid Factor
HGG	Hypergammaglobulinemia
